# In Vitro Biofilm Formation on 3D-Printed, Milled, and Conventionally Manufactured Denture Base Resins

**DOI:** 10.3390/bioengineering13040424

**Published:** 2026-04-03

**Authors:** Michael del Hougne, Alexander Mitzscherling, Andrea Ewald, Tatjana Schilling, Philipp Stahlhut, Uwe Gbureck, Marc Schmitter

**Affiliations:** 1 Department of Prosthodontics, University of Würzburg, Pleicherwall 2, 97070 Würzburg, Germanyschmitter_m@ukw.de (M.S.); 2 Department for Functional Materials in Medicine and Dentistry, University of Würzburg, Pleicherwall 2, 97070 Würzburg, Germany; andrea.ewald@uni-wuerzburg.de (A.E.); philipp.stahlhut@uni-wuerzburg.de (P.S.); uwe.gbureck@uni-wuerzburg.de (U.G.)

**Keywords:** biofilm, dental, dental prosthesis, CAD/CAM, 3D printing, additive manufacturing, subtractive manufacturing, biofilm formation

## Abstract

Biofilm formation on denture base materials may contribute to oral diseases such as denture stomatitis and therefore represents an important factor in prosthodontic treatment. This in vitro study investigated biofilm formation on dental prosthetic materials manufactured by additive, subtractive, and conventional techniques. Disc-shaped specimens were fabricated from 3D-printed Denture Base Resin (Formlabs), milled Lucitone Digital Fit (Dentsply Sirona), and conventionally processed cold-polymerized PALAPress (Kulzer). Biofilm formation by *Streptococcus mutans* and *Streptococcus sanguinis* was assessed separately over a 21-day incubation period using crystal violet staining and photometric determination of optical density at eight predefined time points. Surface characteristics before and after microbial colonization were qualitatively evaluated by scanning electron microscopy. For *S. mutans*, significant material-dependent differences were observed only at selected time points, while overall biofilm accumulation remained low. In contrast, *S. sanguinis* exhibited pronounced and repeated differences, with milled PMMA generally showing lower biofilm accumulation compared with additively manufactured and conventionally processed materials. Overall, *S. sanguinis* formed significantly more biofilm than *S. mutans* across all materials and time points. These findings indicate that both manufacturing technique and bacterial species influence biofilm formation on denture base materials.

## 1. Introduction

In cases of partial or complete tooth loss, treatment often involves removable partial dentures (RPD) or complete dentures (CD), and the prevalence of complete edentulism remains considerable [1]. These prostheses may be used on a temporary or permanent basis. A wide variety of polymers is utilized for various applications in prosthodontics; however, for the fabrication of denture teeth and bases and the repair of dental prostheses, polymethyl methacrylate (PMMA) is the most commonly used material [2,3]. In conventional denture fabrication, PMMA-based materials are typically processed using a powder–liquid system consisting of a polymer powder and a liquid monomer containing cross-linking agents and inhibitors. Polymerization proceeds via a free-radical addition reaction of methyl methacrylate (MMA), resulting in the formation of polymethyl methacrylate [3]. With the increasing digitalization of dentistry, novel manufacturing approaches have enabled additive and subtractive computer-aided design and manufacturing (CAD/CAM) techniques. Subtractive manufacturing by milling is a long-established and clinically proven technique, whereas additive manufacturing (3D printing) has rapidly increased in importance and continues to broaden its range of clinical applications [4,5]. In 3D printing, unpolymerized resin materials are processed and polymerized layer by layer using a light-based curing process. Compared to conventional fabrication, milled dentures have an improved biocompatibility, fit and mechanical properties due to the manufacturing process, resulting in a higher degree of retention and improved mechanical properties [6,7].

Oral surfaces are rapidly colonized by complex microbial biofilms dominated by streptococcal species, which act as typical early bacterial representatives in the oral cavity [8]. These pioneer organisms, with *Streptococcus sanguinis* representing a typical early colonizer and *Streptococcus mutans* being a key biofilm-associated species, play a crucial role in the initial adhesion to oral and prosthetic surfaces and strongly influence subsequent biofilm maturation and microbial succession [9,10]. Oral biofilms are closely associated with a range of biofilm-related diseases, including caries, periodontal diseases, and denture-associated mucosal inflammation such as denture stomatitis [11]. Denture stomatitis is a common inflammatory condition associated with denture use, in which plaque biofilm accumulation induces a localized inflammatory reaction characterized by erythema and, in some cases, mucosal hyperplasia [12]. The prevalence of denture stomatitis is particularly high among elderly and medically compromised patients, including individuals with systemic diseases, immunosuppression, or reduced salivary flow [13].

In edentulous patients, complex biofilm communities persist on both mucosal tissues and denture surfaces, emphasizing the clinical relevance of biofilm formation even in the absence of natural teeth [14]. Early colonizers contribute both to microbial homeostasis and to pathogenic shifts by modulating biofilm structure, metabolic activity, and interspecies interactions [9,10]. Oral biofilms on denture surfaces can also include fungal species, especially Candida albicans. Candida albicans is frequently associated with denture-associated stomatitis and polymicrobial denture biofilms [15]. Nevertheless, the present study focused deliberately on early bacterial colonizers to investigate species-specific biofilm formation under controlled in vitro conditions.

However, the influence of different denture base materials and manufacturing techniques on bacterial adhesion and biofilm formation remains unclear. The null hypothesis of this study was that the manufacturing method of denture base materials (additive, subtractive, or conventional processing) has no effect on the extent of biofilm formation by *Streptococcus mutans* and *Streptococcus sanguinis*.

## 2. Materials and Methods

### 2.1. Specimen Fabrication

**Figure 1:**
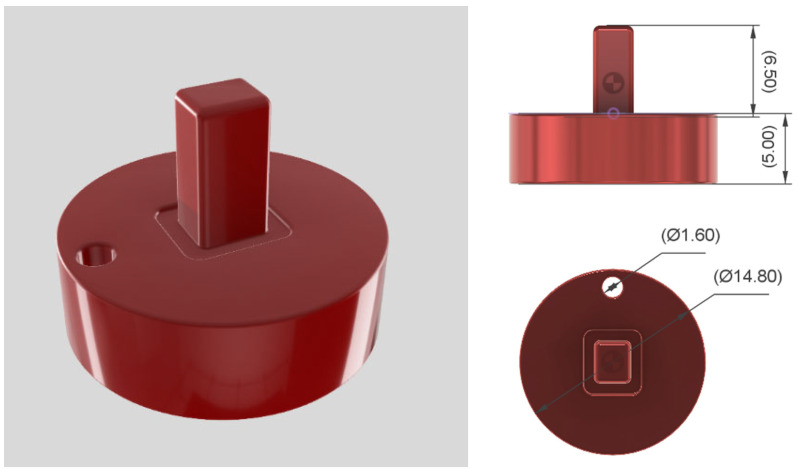
Schematic visualization of specimens and specimen geometry with dimensions in millimeters.

Disc-shaped specimens were designed with a diameter of 14.8 mm and a thickness of 5 mm with Autodesk Fusion 360 (v.2.0.18441, Autodesk Inc., San Francisco, CA, USA). Additionally, the specimens had a bore of 1.6 mm and a rectangular-shaped attachment for improved handling, as shown in Figure 1.

The denture base materials investigated in this study differed not only in their manufacturing technique but also in their chemical formulation, as declared by the respective manufacturers. The chemical compositions of all materials, as detailed in Table 1, were compiled based on the information provided in the corresponding safety data sheets. However, due to proprietary formulations, the level of detail varied between materials, and exact concentrations were not disclosed in all cases.

A Form 3B (Formlabs GmbH, Berlin, Germany) 3D printer with Denture Base OP Resin (RS-F2-DBOP-01, Formlabs GmbH, Berlin, Germany) was utilized for the additively manufactured specimens. The CAD files were transferred to the printing software PreForm (version 3.39.1, Formlabs GmbH, Berlin, Germany) and aligned such that the flat surface was parallel to the build platform. A Z-axis resolution of 0.05 mm was chosen. The post-processing, according to the manufacturer’s instructions, included washing with Form Wash (Formlabs GmbH, Berlin, Germany) with isopropanol for 15 min, airdrying for 30 min and post-curing with Form Cure (Formlabs GmbH, Berlin, Germany) in glycerol for an additional 60 min at 80 °C. Afterwards, support structures were removed. Subtractively manufactured specimens were dry milled with inLab MC X5 (Dentsply Sirona Deutschland GmbH, Bensheim, Germany) and Lucitone Digital Fit (D906110, Dentsply Sirona Deutschland GmbH, Bensheim, Germany) blanks colored in “original” with a diameter of 98 mm and a height of 20 mm. The CAD files were processed with inLab CAM Software (version 22.7.1.284899, Dentsply Sirona Deutschland GmbH, Bensheim, Germany) and the specimens were nested with a parallel orientation to the blank. Milling was conducted with the production quality set to “high”. Conventionally processed cold-polymerized PMMA specimens were fabricated using a putty mold (Virtual Putty fast, Ivoclar Vivadent GmbH, Ellwangen, Germany) derived from a CAM-manufactured reference specimen. PALAPress (Kulzer GmbH, Hanau, Germany) was used for polymerization and processed according to manufacturer’s instructions with 10 g polymer powder and 7 mL monomer liquid. The specimens were stored under pressure at 45 °C and 2 bars for 15 min.

A standardized finishing and polishing protocol was used for the specimens. Polishing was performed on a rotating disc under continuous water cooling using gentle contact pressure and a Buehler MetaServ 3000 (Buehler, ITW Test & Measurement GmbH, Leinfelden-Echterdingen, Germany) grinding and polishing machine. The initial abrasive grit differed according to the material type. For additively manufactured and milled PMMA specimens, grinding was performed sequentially using silicon carbide wet abrasive papers with grit sizes P500, P1200, and P4000. In contrast, conventionally fabricated cold-polymerized PMMA specimens required an initial coarse grinding step due to their undefined surface, starting with P80, followed by P500, P1200, and P4000. Subsequent polishing was carried out utilizing a cotton buff and polishing liquid (KMG Liquid, Candulor AG, Opfikon, Switzerland). The reverse side of each specimen was ground using fine dental corundum paper (grit size 320, Finocorund Plus, FINO GmbH, Kassel, Germany). Finally, all specimens were cleaned on both sides with a steam cleaner (Triton SLA, Bego GmbH & Co., KG, Bremen, Germany). The polished surface was considered the standardized reference surface for surface evaluation and SEM observation, whereas the reverse side represented a less highly finished surface in partial analogy to the tissue-contacting intaglio surface of a denture. This approach was chosen to reflect clinically relevant differences between polished and non-polished denture surfaces while maintaining a defined and reproducible observation surface.

### 2.2. Biofilm Formation and Quantification

**Figure 2:**
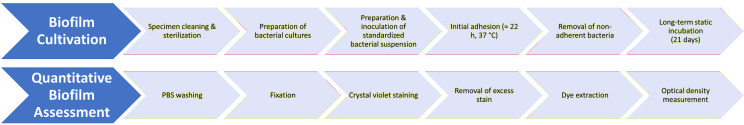
Systematic flowchart of biofilm cultivation and quantitative assessment by crystal violet staining and optical density measurement.

Biofilm formation was investigated using *Streptococcus mutans* (DSM 20523) and *Streptococcus sanguinis* (DSM 20567). The experimental workflow is summarized in Figure 2. For the quantitative biofilm analysis, three independent specimens were included per material, bacterial species, and measurement time point. Each specimen was assessed by duplicate photometric measurements after crystal violet staining. Overall, 144 specimens were analyzed in the quantitative assay. This sample size was determined a priori according to the exploratory nature of the in vitro study and practical feasibility, while maintaining biological triplicates for all experimental conditions.

For *S. sanguinis*, incubation and biofilm cultivation were conducted under strictly anaerobic conditions throughout the entire experimental period to meet the species-specific growth requirements. Bacterial strains were revived from glycerol stocks and cultured overnight in tryptic soy broth supplemented with yeast extract (TSY) at 37 °C. From fresh agar plates, single colonies were used to prepare overnight cultures, which were subsequently adjusted by serial dilution to a standardized starting concentration of approximately 5 × 10^2^ CFU/mL in fresh medium. Specimens were subjected to ultrasonic cleaning in 0.9% sodium chloride solution for 30 min at 40 °C. This ultrasonic cleaning was performed twice, each time using a fresh 0.9% sodium chloride solution. Subsequently, specimens were immersed in 70% ethanol for 15–20 min, turned once to ensure complete surface exposure, and the ethanol was then aspirated. Finally, specimens were air-dried under a sterile laminar airflow cabinet. The sterile specimens of each material type as well as glass coverslips (positive controls) were placed individually into sterile 24-well plates. Each well was inoculated with 1.5 mL of standardized bacterial suspension, while material controls without bacterial inoculation served as negative controls. During incubation, all specimens were placed in a standardized orientation with the polished reference surface facing upward to ensure consistent exposure conditions for biofilm formation. Following inoculation, specimens were incubated at 37 °C for approximately 22 h to allow initial bacterial adhesion. After 16 h of incubation, non-adherent bacteria were removed by carefully aspirating the supernatant. Specimens were gently transferred into wells containing sterile 0.9% NaCl for washing and subsequently into fresh wells containing growth medium. Biofilm cultivation was continued under static conditions at 37 °C for a total period of 21 days. During the incubation period, the growth medium was exchanged at predefined intervals by aspirating the supernatant and replenishing each well with 1.5 mL of fresh medium under sterile conditions. Biofilm quantification was performed on eight predefined measurement days (days 1, 2, 5, 7, 9, 12, 14, and 21). At each measurement time point, the culture medium was aspirated and specimens were gently washed with phosphate-buffered saline (PBS) to remove non-adherent bacteria. Biofilms were fixed with 4% paraformaldehyde for 10 min, washed with PBS, and stained with 0.1% crystal violet solution for 20 min at room temperature. Excess stains were removed by repeated washing with 0.9% NaCl. The crystal violet bound to the biofilm was subsequently extracted using 96% ethanol under gentle agitation for 45 min. Portions of the resulting dye solution were transferred in duplicate to a 96-well plate, and the optical density (OD) was measured at 570 nm using a microplate reader (Spark 20 M, Tecan Trading AG, Männedorf, Switzerland) with SparkControl software (version 1.2.25, Tecan Trading AG, Männedorf, Switzerland), with 96% ethanol serving as the blank. Material-specific negative control specimens without bacterial inoculation were processed identically, and the corresponding blank values were subtracted from the measured OD values to correct for background absorption. For each bacterial strain, measurement day, and material type, three independent specimens were analyzed, and each extracted dye solution was measured in duplicate, resulting in six measurement values per condition. The corrected OD values were considered proportional to the amount of biofilm biomass present on the specimen surfaces. The culture medium was replaced every 48 h throughout the experiment. To ensure undisturbed biofilm development prior to analysis, the wells scheduled for the next measurement were not subjected to medium replacement during the preceding 48 h. Routine contamination checks were performed by plating aliquots of the culture supernatant on agar plates followed by incubation and visual inspection. Furthermore, all experimental procedures were performed under sterile conditions, with separate handling of bacterial strains and the use of freshly prepared overnight cultures for each experimental run to prevent cross-contamination.

### 2.3. Scanning Electron Microscopy Analysis

**Figure 3:**
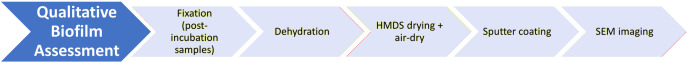
Systematic flowchart of qualitative biofilm assessment with SEM.

Secondary electron images were taken with a field emission electron microscope (Crossbeam 340, Carl Zeiss Microscopy Deutschland GmbH, Oberkochen, Germany) at an acceleration voltage of 2 kV to qualitatively assess surface topography and biofilm morphology before and after microbial colonization. The experimental workflow is summarized in Figure 3. Specimens from each material group were examined prior to incubation and following biofilm formation to evaluate material-specific surface characteristics and the structural organization of the adherent biofilms. SEM analysis after microbial colonization was performed after 12 days of incubation as a representative later-stage time point to qualitatively assess established biofilm morphology. For scanning electron microscopy (SEM) analysis after microbial colonization following fixation, specimens were dehydrated to preserve biofilm structure and prevent collapse. Dehydration was performed using a graded ethanol series, starting with incubation in PBS on ice, followed by sequential incubation in 70%, 90%, and 100% ethanol at room temperature. Specimens were then dried using hexamethyldisilazane (HMDS). Samples were immersed twice in HMDS for 15 min, after which the reagent was carefully removed, and specimens were air-dried under ambient conditions. specimens were sputter-coated with a thin layer of platinum using a sputter coater (EM ACE600, Leica Microsystems GmbH, Wetzlar, Germany).

### 2.4. Statistical Analysis

Statistical analysis was performed using SPSS software (V.29, IBM SPSS Statistics, IBM Corp., Armonk, NY, USA). The level of significance was set at *p* < 0.05. Due to the small sample size per group, nonparametric methods were applied. Differences in biofilm formation between material groups at each measurement day were analyzed using the Kruskal–Wallis test followed by Dunn’s post hoc test with Bonferroni correction for multiple comparisons. Effect sizes (η^2^) were calculated from the Kruskal–Wallis H statistic using η^2^ = (H − k + 1)/(N − k). Post hoc statistical power (1 − β) was estimated using G*Power (version 3.1.9.7, Heinrich-Heine-University Düsseldorf, Düsseldorf, Germany) based on the corresponding effect size f derived from η^2^. Comparisons between the bacterial species were conducted using the Mann-Whitney U test.

## 3. Results

### 3.1. Quantitative Assessment with OD

The results of the corrected OD measurements are summarized in Table 2.

**Figure 4:**
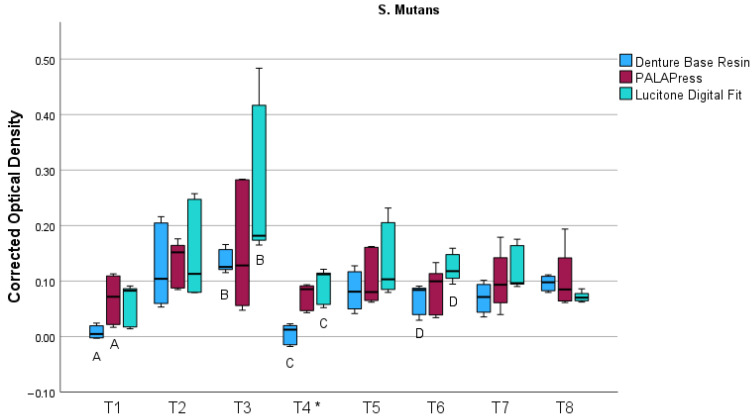
Boxplot of corrected optical density at 570 nm measured on eight predefined time points (T1-T8 corresponding to days 1, 2, 5, 7, 9, 12, 14, and 21) for *Streptococcus mutans* biofilms formed on Denture Base Resin, PALAPress, and Lucitone Digital Fit specimens. The data points at T4 were affected by contamination and thus marked with an asterisk (*). Different capital letters indicate statistically significant differences (*p* < 0.05) between different materials.

For *S. mutans*, the results are visualized in a boxplot in Figure 4. On T4, contamination occurred in the *S. mutans* samples, rendering the corresponding measurement unreliable. The contamination observed at this time point was macroscopically visible and likely represented fungal growth. This data point was therefore not considered in the statistical evaluation and is marked with an asterisk in the boxplot for transparency. Overall, biofilm formation was low, with the highest mean value observed for Lucitone Digital Fit after 5 days (0.27 ± 0.14). As summarized in Table 3, the Kruskal–Wallis test revealed significant differences between the investigated materials for *S. mutans* at selected time points (T1, T3, and T6), with moderate to very large effect sizes.

Pairwise comparisons using Dunn’s post hoc test revealed significant differences after 1 day between 3D-printed and conventionally fabricated specimens (*p* = 0.045). After 5 days (*p* = 0.015) and 12 days (*p* = 0.004), significant differences occurred between 3D-printed and milled PMMA specimens. Overall, milled specimens generally had higher corrected OD values compared with 3D-printed materials.

**Figure 5:**
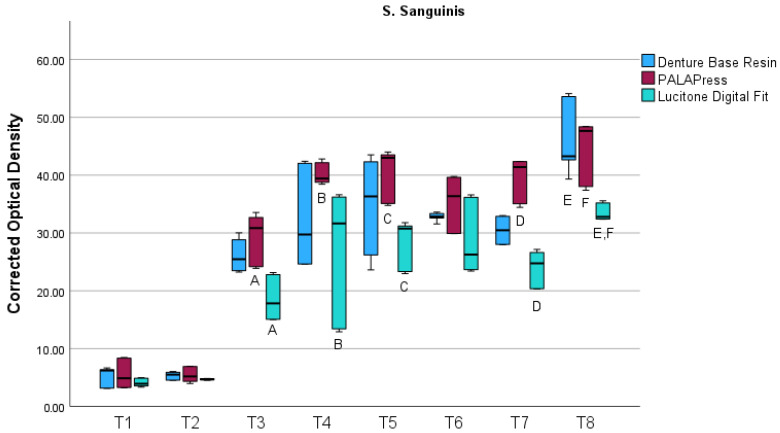
Boxplot of corrected optical density at 570 nm measured on eight predefined time points (T1-T8 corresponding to days 1, 2, 5, 7, 9, 12, 14, and 21) for *Streptococcus sanguinis* biofilms formed on Denture Base Resin, PALAPress, and Lucitone Digital Fit specimens. Different capital letters indicate statistically significant differences (*p* < 0.05) between different materials.

The results of the corrected OD measurements for *S. sanguinis* are visualized in a boxplot in Figure 5. For *S. sanguinis*, biofilm formation exhibited a non-linear temporal pattern across all materials. An initial increase was observed up to the mid-experimental period, with intermediate peak values around day 7 and day 9, followed by a transient reduction at subsequent time points and a pronounced final increase toward day 21.

As summarized in Table 4, the Kruskal–Wallis test revealed significant differences between the investigated materials for *S. sanguinis* at selected time points (T3, T4, T5, T7 and T8), with effect sizes ranging from moderate to very large.

Pairwise comparisons using Dunn’s post hoc test revealed significant differences between milled and conventionally fabricated specimens were observed after 5 days (*p* = 0.002), 7 days (*p* = 0.011), 9 days (*p* = 0.012) and 14 days (*p* < 0.001). After 21 days, milled specimens had significantly less biofilm than 3D-printed specimens (*p* = 0.007) and conventionally fabricated specimens (*p* = 0.015). Overall, milled PMMA had less biofilm than 3D-printed and conventionally fabricated specimens, while the 3D-printed and conventional groups generally exhibited higher and more variable biofilm accumulation at later time points.

When comparing biofilm formation between both bacterial species, *S. sanguinis* exhibited significantly higher biofilm accumulation than *S. mutans* across all materials and measurement time points (*p* = 0.002).

The null hypothesis stated that the manufacturing method of denture base materials would have no effect on the extent of biofilm formation by *Streptococcus mutans* and *Streptococcus sanguinis*. Based on the results, the null hypothesis could not be fully accepted. While the influence of the manufacturing method on *S. mutans* biofilm formation was limited and inconsistent, significant and repeated material-dependent differences were observed for *S. sanguinis*, particularly with lower biofilm accumulation on milled PMMA specimens. These findings indicate that the effect of manufacturing technique on biofilm formation is species-specific and more pronounced for *S. sanguinis* than for *S. mutans*.

### 3.2. Qualitative Assessment with SEM

**Figure 6:**
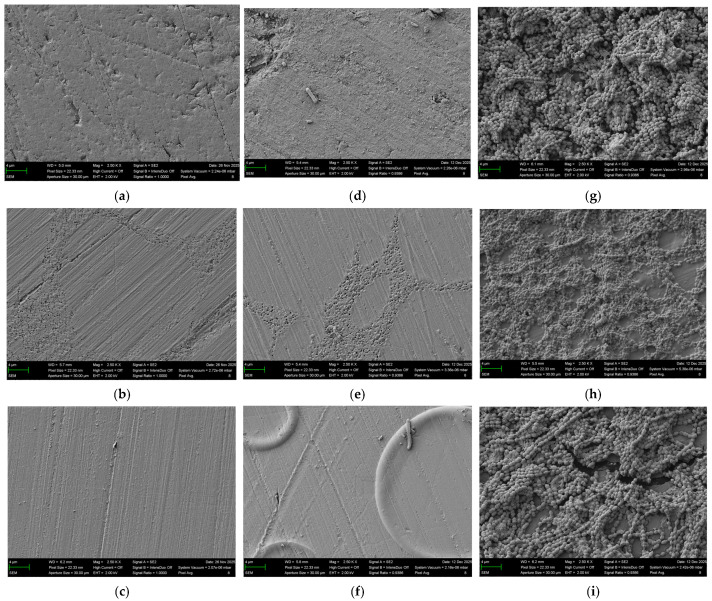
Representative SEM images of specimens at magnification 2500×. (**a**) Denture Base Resin before microbial colonization. (**b**) Lucitone Digital Fit before microbial colonization. (**c**) PALAPress before microbial colonization. (**d**) Denture Base Resin after 12 days of microbial colonization with *S. mutans*. (**e**) Lucitone Digital Fit after 12 days of microbial colonization with *S. mutans*. (**f**) PALAPress after 12 days of microbial colonization with *S. mutans*. (**g**) Denture Base Resin after 12 days of microbial colonization with *S. sanguinis*. (**h**) Lucitone Digital Fit after 12 days of microbial colonization with *S. sanguinis*. (**i**) PALAPress after 12 days of microbial colonization with *S. sanguinis*.

The qualitative differences in surface topography prior to microbial colonization, as well as the distinct patterns of bacterial adhesion and biofilm formation after 12 days of incubation with *S. mutans* and *S. sanguinis*, are illustrated in Figure 6.

A qualitative assessment of the surface topography before microbial colonization of the 3D-printed specimens showed an irregular surface morphology with visible microstructural features attributable to the layer-by-layer fabrication process. The surface appeared non-uniform, with small elevations and depressions distributed across the specimen. The milled specimens had a relatively homogeneous surface morphology with fine linear structures across the surface, attributable to the standardized grinding and polishing procedure. In addition, scattered small, dot-like depressions were observed, resulting in a mildly heterogeneous microtopography. The surface of the conventionally processed PMMA specimens appeared relatively uniform following finishing and polishing with linear polishing marks.

Overall, the SEM images of the specimens after 12 days of incubation revealed structures consistent with bacterial biofilms formed by the investigated species. Although contamination occurred at T4 with *S. mutans* based on agar plating and macroscopic inspection, no morphological features indicating contamination were detected in the SEM images.

In the SEM images of the specimens after 12 days of incubation with *S. mutans* isolated rod-shaped bacterial structures consistent with the morphology of *S. mutans* was observed on the specimen surfaces. The findings were limited to individual bacterial cells, and no continuous or confluent biofilm structures were detected.

The incubation for 12 days with *S. sanguinis* bacterial colonization was more pronounced compared with *S. mutans*. The SEM images revealed numerous coccoid bacterial cells forming clustered aggregates and multilayered structures on the specimen surfaces, indicative of advanced biofilm formation. The extent of biofilm coverage observed in the SEM images qualitatively correlated with the OD measurements, with 3D-printed and conventionally fabricated specimens exhibiting more pronounced bacterial colonization than milled specimens.

## 4. Discussion

### 4.1. Interpretation of the Results

The findings of this study demonstrate a time-dependent pattern of biofilm development for both *S. mutans* and *S. sanguinis*. For *S. mutans*, biofilm accumulation remained generally low throughout the observation period, with only moderate fluctuations over time. This may be related to the culture conditions utilized. The experiments were performed with the protein-rich, glucose-containing medium TSY, which is recommended for both *S. mutans* and *S. sanguinis*. This allowed standardized and reproducible growth conditions. However, sucrose is known to enhance extracellular polysaccharide production via glucosyltransferase activity and thereby promotes the formation of a structured biofilm matrix [16,17]. The addition of sucrose also alters bacterial metabolism and may influence the kinetics and architecture of biofilm development rather than simply increasing total biomass. While the absence of sucrose in the present study ensured controlled experimental conditions, future studies should investigate how sucrose supplementation affects material-dependent differences in biofilm formation under conditions more closely resembling the oral environment. In contrast, *S. sanguinis* showed a descriptive temporal pattern across the investigated time points with varying biomass levels during incubation. This pattern may reflect different stages of biofilm maturation, including initial adhesion, accumulation, structural consolidation, and potential nutrient-related plateau effects. The transient reduction observed after intermediate peaks could be associated with partial detachment phenomena or metabolic adaptation within aging biofilms [18,19]. Overall, biofilms are complex and dynamic as they are living structures and undergo several stages, including aggregation, microcolony formation, maturation, and dispersal [20,21]. *S. sanguinis* formed significantly greater biofilm than *S. mutans*. This difference may be attributed to distinct adhesion mechanisms and extracellular matrix production. *S. sanguinis*, as a typical early colonizer, expresses multiple structures such as surface adhesins and fimbriae [10,22]. Material-related properties likely influenced bacterial adhesion and biofilm accumulation. Although all specimens underwent standardized finishing and polishing to reduce surface-related differences, subtle variations in material-specific characteristics may still have contributed to the observed findings. Surface properties such as roughness and surface free energy are known to affect bacterial adhesion and biofilm development on dental materials—it is reported that increased surface roughness and higher surface energy have been associated with enhanced microbial retention and biofilm formation, whereas smoother surfaces tend to resist initial bacterial attachment [23,24]. However, these parameters were not quantified in the present study. Therefore, the observed differences in biofilm formation are more likely to reflect material-dependent factors such as surface chemistry, internal microstructure, degree of polymerization, or other manufacturing-related characteristics. Moreover, denture base materials manufactured by CAD/CAM milling typically exhibit a higher degree of conversion and lower residual monomer content than conventionally processed PMMA, which may reduce surface reactivity and susceptibility to biofilm accumulation [3]. In contrast, conventionally processed and additively manufactured materials may present minor surface irregularities or microstructural heterogeneities, affecting bacterial retention [25,26]. The evaluation of SEM images of specimens after 12 days of microbial colonization supported the quantitative OD measurements. The absence of foreign morphological structures in SEM images obtained after 12 days of incubation reinforced that the contamination observed at T4 only in the samples designated for measurements of T4. All the samples set up for the other timepoints have been sterile. This is in line with the implemented contamination control measures and indicates that the SEM analysis reflected the intended bacterial. For *S. sanguinis*, specimens with higher OD values generally exhibited more extensive bacterial coverage, whereas milled specimens had less colonization. As SEM only provides descriptive morphological information, despite correspondence with OD data, visual findings should be interpreted cautiously. Arzani et al. found that 3D-printed denture bases showed greater microbial metabolic activity and higher numbers of adherent microorganisms than those fabricated by milling. This difference was likely related to material characteristics associated with additive manufacturing, such as polymerization behavior and residual monomer content. Meta-regression analysis indicated that surface roughness alone did not significantly influence microbial outcomes, suggesting that chemical and structural properties of the material play a more important role in microbial colonization than surface texture [27]. Alqarawi et al. also found that fabrication methods of denture base materials significantly affected the microbial adhesion in their study, with low microbial adhesion for milled materials [28]. Alqarawi et al. concluded that improvements to the antiadherent properties of 3D-printed resins could be obtained by incorporating antifungal agents or changing the printing parameters. Other studies also found increased candida albicans adhesion to 3D-printed materials and rough surface topographies, compared to milled materials. These findings are consistent with the outcomes of the present study and highlight the material- and fabrication-specific nature of biofilm formation.

### 4.2. Methodological Considerations

The crystal violet staining method for biofilm is widely used, cost-effective, and suitable for comparative analyses across different materials [29,30]. This method allows semiquantitative assessment of total biofilm biomass and has been extensively validated in vitro biofilm research. However, this method does not differentiate between viable and non-viable cells or distinguish between cellular and extracellular material, and therefore does not provide specific information on microbial viability or composition [31,32]. For the interpretation of biomass readings obtained by crystal violet staining, these limitations should be considered, especially for complex or multispecies biofilm models. Alternative analytical approaches including fluorescence-based methods were tested by the authors prior to the crystal violet straining method. However, due to pronounced material-related autofluorescence, particularly of the 3D-printed resin, interference with the signal interpretation and precluded its use as a reliable methodology. Material-related background absorption constitutes an important methodological consideration when optical density-based measurements are used. PMMA-based materials may exhibit intrinsic absorbance or nonspecific dye binding, making the inclusion of material-specific negative controls essential [33]. Accordingly, blank specimens without bacterial inoculation were processed in parallel, and their optical density values were consistently subtracted from the measured data to correct for background signals and enhance data reliability. Additional methods such as LIVE/DEAD staining or real-time polymerase chain reaction (qPCR) could have provided complementary information [34,35]. These methods would have required separate experimental series as they cannot be applied to the same specimens. The fluorescence-based LIVE/DEAD assay was not suitable in this study due to the autofluorescence of the tested materials. The qPCR method would reflect gene expression rather than quantifying the amount of extracellular matrix deposited on the specimen surfaces. However, the number of specimens per group was limited due to practical feasibility. Although triplicate specimens with duplicate measurements were used to enhance reproducibility, the limited sample size may restrict the detection of subtle effects. Nevertheless, the applied protocol allowed consistent trend analysis across materials and time points, providing a reliable basis for comparative evaluation. Surface finishing and polishing were standardized to minimize variability and enhance comparability between materials. While this approach improves experimental reproducibility, it may only partially reflect clinical conditions, where polishing quality and wear can vary considerably.

### 4.3. Limitations and Future Perspectives

The present study employed monoculture biofilm models, representing a simplified experimental system. This enabled controlled investigation of species-specific biofilm behavior. However, it does not reflect the complexity of the oral environment with multispecies biofilm development through dynamic interbacterial interactions, and other relevant factors such as the influence of salivary proteins, shear forces and relationships between microorganisms [36]. Several oral environmental conditions known to affect biofilm formation were not simulated, including the presence of saliva, temperature fluctuations, pH variations, and mechanical cleaning. The absence of these parameters limits the direct transferability of the present findings to clinical conditions [36]. Despite these limitations, the results provide valuable insights into material- and species-dependent differences in biofilm formation on denture base materials. The observed differences may nonetheless be relevant for material selection and the development of effective hygiene strategies, particularly in patients with limited oral hygiene capability due to advanced age or motor impairments. Future studies should include additional species such as Candida albicans, multispecies biofilm models, and saliva-derived components. These modifications would increase the physiological relevance of the experimental setting and better reflect the complexity of the oral environment, where fungal colonization and salivary pellicle formation can substantially impact microbial adhesion and biofilm development on dental materials. In addition, in vivo validation is required to confirm the present findings under clinical conditions. Further investigations should also address the effects of material aging, routine cleaning procedures, and surface modifications or antimicrobial coatings on biofilm formation and long-term clinical performance.

## 5. Conclusions

Within the limitations of this in vitro study, both the manufacturing technique of denture base materials and the bacterial species significantly influenced biofilm formation. Biofilm accumulation by *S. mutans* was generally low and only sporadic material-dependent differences were observed, indicating a limited sensitivity to the manufacturing method. In contrast, *S. sanguinis* demonstrated pronounced, time-dependent, and material-specific differences, with milled PMMA specimens consistently exhibiting lower biofilm accumulation than additively manufactured and conventionally fabricated specimens. Across all materials and time points, *S. sanguinis* formed substantially more biofilm than *S. mutans*, highlighting species-specific biofilm behavior. Qualitative SEM analysis supported the quantitative findings by revealing material-dependent differences in surface topography and biofilm morphology after microbial colonization. Although the present findings suggest potential advantages of milled PMMA in reducing biofilm accumulation by early colonizing bacteria, their clinical relevance remains to be determined. Future studies should incorporate multispecies biofilm models, dynamic oral conditions, and in vivo approaches to reflect the complex biological environment of the oral cavity and to validate the observations of this in vitro study.

## Figures and Tables

**Table 1:** Manufacturer-declared chemical composition of the denture base materials investigated, as reported in the corresponding safety data sheets. Exact concentrations were not disclosed for all materials due to proprietary formulations.

Material	Main Polymer Matrix	Monomers/Cross-Linkers	Additives/Initiators	Composition
Denture Base OP Resin, RS-F2-DBOP-01 (Formlabs GmbH, Berlin, Germany)	Methacrylate-based resin	Urethane dimethacrylate; Propylidynetrimethyl trimethacrylate	Diphenyl(2,4,6-trimethylbenzoyl)phosphine oxide (photoinitiator)	Methacrylate monomers; urethane dimethacrylate; propylidynetrimethyl trimethacrylate; diphenyl(2,4,6-trimethylbenzoyl)phosphine oxide
Lucitone Digital Fit Original, 20 mm, D906110, (Dentsply Sirona Deutschland GmbH, Bensheim, Germany)	PMMA-based polymer matrix	Residual methyl methacrylate	Titanium dioxide (pigment)	Methyl methacrylate < 1%; Titanium dioxide < 0.5%
PALApress (Kulzer GmbH, Hanau, Germany)	Acrylic polymer (PMMA)	Methyl methacrylate; butanediol dimethacrylate; pentaerythritol tetraacrylate	Dibenzoyl peroxide; hydroquinone monomethyl ether; pigments	proprietary formulation

**Table 2:** Results of optical density measurements presented as mean ± standard deviation.

Day	Material	*S. mutans*	*S. sanguinis*
1	Denture Base Resin	0.008 ± 0.012	5.287 ± 1.669
	Lucitone Digital Fit	0.062 ± 0.036	4.098 ± 0.675
	PALA Press	0.067 ± 0.041	5.512 ± 2.357
2	Denture Base Resin	0.123 ± 0.071	5.340 ± 0.661
	Lucitone Digital Fit	0.148 ± 0.083	4.691 ± 0.118
	PALA Press	0.136 ± 0.040	5.430 ± 1.265
5	Denture Base Resin	0.135 ± 0.021	26.075 ± 2.827
	Lucitone Digital Fit	0.267 ± 0.144	18.621 ± 3.600
	PALA Press	0.154 ± 0.105	29.326 ± 4.259
7	Denture Base Resin	0.006 ± 0.018	32.197 ± 8.098
	Lucitone Digital Fit	0.095 ± 0.031	27.078 ± 11.007
	PALA Press	0.074 ± 0.023	40.154 ± 1.864
9	Denture Base Resin	0.083 ± 0.035	34.712 ± 8.197
	Lucitone Digital Fit	0.135 ± 0.066	28.464 ± 4.145
	PALA Press	0.108 ± 0.047	40.561 ± 4.380
12	Denture Base Resin	0.069 ± 0.027	32.792 ± 0.724
	Lucitone Digital Fit	0.124 ± 0.025	28.724 ± 6.062
	PALA Press	0.087 ± 0.041	35.319 ± 4.460
14	Denture Base Resin	0.070 ± 0.026	30.467 ± 2.219
	Lucitone Digital Fit	0.120 ± 0.039	23.991 ± 3.150
	PALA Press	0.101 ± 0.052	39.601 ± 3.315
21	Denture Base Resin	0.096 ± 0.013	46.029 ± 6.248
	Lucitone Digital Fit	0.072 ± 0.009	33.523 ± 1.466
	PALA Press	0.105 ± 0.052	44.581 ± 5.338

**Table 3:** Significant Kruskal–Wallis test results for *S. mutans* with corresponding effect sizes (η^2^) and post hoc power (1 − β).

Time Point	Significance (*p*)	η^2^	1 − β
T1	0.025	0.360	0.245
T3	0.036	0.309	0.184
T6	0.016	0.415	0.328

**Table 4:** Significant Kruskal–Wallis test results for *S. sanguinis* with corresponding effect sizes (η^2^) and post hoc power (1 − β).

Time Point	Significance (*p*)	η^2^	1 − β
T3	0.002	0.703	0.932
T4	0.032	0.326	0.203
T5	0.016	0.419	0.336
T7	0.001	0.877	1.000
T8	0.003	0.628	0.795

## Data Availability

The original contributions presented in this study are included in the article. Further inquiries can be directed to the corresponding author.

## Abbreviations

The following abbreviations are used in this manuscript:

3D	Three-dimensional
CAD/CAM	Computer-aided design/computer-aided manufacturing
CD	Complete denture
CFU	Colony-forming units
DSM	Deutsche Sammlung von Mikroorganismen (German Collection of Microorganisms and Cell Cultures)
HMDS	Hexamethyldisilazane
MMA	Methyl methacrylate
OD	Optical density
PBS	Phosphate-buffered saline
PMMA	Polymethyl methacrylate
qPCR	real-time polymerase chain reaction
RPD	Removable partial denture
SEM	Scanning electron microscopy
TSY	Tryptic soy broth supplemented with yeast extract
wt%	Weight percent
